# The roles of call wall invertase inhibitor in regulating chilling tolerance in tomato

**DOI:** 10.1186/s12870-017-1145-9

**Published:** 2017-11-09

**Authors:** Xiao-xia Xu, Qin Hu, Wan-nian Yang, Ye Jin

**Affiliations:** Hubei Key Laboratory of Genetic Regulation and Integrative Biology, School of Life Sciences, Central China Normal University, Wuhan, 430079 People’s Republic of China

**Keywords:** Abscisic acid, Cell wall invertase inhibitor, Chilling tolerance, C-repeat binding factors, *Solanum lycopersicum*, Sugar signaling

## Abstract

**Background:**

Hexoses are important metabolic signals that respond to abiotic and biotic stresses. Cold stress adversely affects plant growth and development, limiting productivity. The mechanism by which sugars regulate plant cold tolerance remains elusive.

**Results:**

We examined the function of INVINH1, a cell wall invertase inhibitor, in tomato chilling tolerance. Cold stress suppressed the transcription of *INVINH1* and increased that of cell wall invertase genes, *Lin6* and *Lin8* in tomato seedlings. Silencing *INVINH1* expression in tomato increased cell wall invertase activity and enhanced chilling tolerance. Conversely, transgenic tomatoes over-expressing *INVINH1* showed reduced cell wall invertase activity and were more sensitive to cold stress. Chilling stress increased glucose and fructose levels, and the hexoses content increased or decreased by silencing or overexpression *INVINH1*. Glucose applied in vitro masked the differences in chilling tolerance of tomato caused by the different expressions of *INVINH1*. The repression of *INVINH1* or glucose applied in vitro regulated the expression of C-repeat binding factors (*CBFs*) genes. Transcript levels of *NCED1*, which encodes 9-cisepoxycarotenoid dioxygenase (NCED), a key enzyme in the biosynthesis of abscisic acid, were suppressed by INVINH1 after exposure to chilling stress. Meanwhile, application of ABA protected plant from chilling damage caused by the different expression of *INVINH1*.

**Conclusions:**

In tomato, INVINH1 plays an important role in chilling tolerance by adjusting the content of glucose and expression of *CBF*s.

**Electronic supplementary material:**

The online version of this article (10.1186/s12870-017-1145-9) contains supplementary material, which is available to authorized users.

## Background

In higher plants, sucrose is the major transport form of carbohydrates. Cleavage of sucrose is catalyzed by either sucrose synthase (EC 2.4.1.13) or invertase (EC 3.2.1.26). The products (hexoses) are not only substrates of respiration and biosynthesis, but also important metabolic signals in plant response to abiotic and biotic stresses [1–5].

Invertases irreversibly hydrolyze sucrose into glucose and fructose. Based on their pH optima, solubility characteristics and subcellular localization, invertases are categorized as vacuolar, neutral/alkaline and cell wall invertases [6–9]. Both vacuolar and neutral/alkaline invertases are soluble, with an acidic pI, while cell wall invertases are insoluble, with a mostly basic pI. Unlike the enzymes in vacuoles or cell walls, neutral/alkaline invertases are not glycosylated and possess an optimal pH of 7.0–7.8 [8, 9]. Neutral/alkaline invertases, localized in cytoplasm and mitochondria [10], are essential for normal plant growth, development and stress responses. Vacuolar invertases, with an optimal pH of 4.7–5.5, correlate with the sugar accumulation in sink tissues [11, 12] and cell expansion [13, 14]. Cell wall invertases, with an optimal pH of 4.3–5.5, hydrolyze sucrose to maintain the sucrose concentration gradient between source and sink tissues [15]. Much progress has been made in understanding the role of cell wall invertase in sink tissue (seed & fruit) development [16–20], in fruit-set under heat stress [21] or during water deficit [6], and in leaf senescence [22].

The protein of cell wall invertase is intrinsically stable because of their glycosylated nature [6, 23]. Thus, the activity of cell wall invertase is largely regulated at the protein level. Inhibitors directly target the invertase active site and compete with sucrose, the substrate of invertase, for the same binding site [24]. After the initial biochemical characterization [25], a group of small proteins (<20 KD) were observed to inhibit the activity of invertase in tobacco [26], maize [27], tomato [18, 28], potato [29], soybean [30] and Arabidopsis [31].

Transgenic approaches have led to some progress in understanding the role of invertase inhibitors in plants. Overexpression of an invertase inhibitor in potato prevented cold-induced sweetening of potato tubers [12, 32, 33]. Suppressing the expression of cell wall invertase inhibitor led to an increase of seed weight in soybean [30] and seed germination in Arabidopsis [31]. Jin et al. [18] demonstrated that *INVINH1* (a cell wall invertase inhibitor) could regulate the activity of the cell wall invertase in vivo, and silencing the expression of *INVINH1* in tomato resulted in enlarged seed size, increased sugar content in fruit and delayed leaf senescence. These studies focused on the function of INVINH1 in sink tissues rather than in vegetative organs, although INVINH1 was highly expressed in root, stem and leaf during the vegetative period.

Interestingly, the expression of the cell wall invertase inhibitor was induced by abscisic acid [18, 23, 30], which is involved in the response to various biotic and abiotic stresses, including chilling stress [34]. Low temperature is an important factor which affects the growth and development of plants. Plants adjust the delicate balance between multiple pathways, including transcription factor, DNA modification, hormones, secondary messengers, phosphatases and protein kinases among others to get acclimatized [35]. The content of sugar, known to have an osmoprotective function increased during cold treatment [36]. Glucose induced the expression of cold response genes [37]. The ectopic expression of tomato GDP-L galactose phosphorylase gene in tobacco enhanced tolerance to chilling stress [38]. These above suggested that sucrose metabolism may be involved in the regulation of chilling tolerance. However little was known about the function of cell wall invertase activity in chilling stress tolerance. This study aimed to explore the roles of INVINH1 in tomato cold tolerance and our data highlight the function of INVINH1 in plant tolerance to cold stress and provide a possible mechanism of plant cold tolerance.

## Methods

### Plant material, growth conditions and cold treatments

Tomatoes (*Solanum lycopersicum* XF-2) were grown in the greenhouse with 16 h of light at 25–27°C and 8 h of darkness at 22°C. Under this conditions the visible flower buds appear 65–75 days after germinate, so we chose 45 days old and 60 days old plants in this study. The first mature leaf was used for RT-PCR, proline content and the peroxidase (POD) activities measurement. For plants grown in vitro, seeds were surface sterilized and germinated on half-strength MS medium without sugar at 25°C with a 16-h photoperiod. For cold treatments, plants were transferred from 25°C to 4°C and were maintained under the same photoperiod as previously described.

### Gene constructs and plant transformation

To construct Rbcs3a:INVINH1, the full-length Rbsc3a promoter [39] was digested with XbaI and HindIII and cloned into vector pCAMBIA1300 (Cambia), upstream of *INVINH1*.

Tomato plants were transformed with Rbcs3a:INVINH1 constructs according to Jin et al. [18]. PCR analysis was used to monitor the incorporation of the transgene. Plants transformed with Rbcs3a:INVINH1 were analyzed using the following primer pair:5′-GCCTCTAGATATTGCTTTCTAGTCTCT-3′ and 5′-GAATTCCAATAAATTTCTTACAAT-3′. Twenty-four primary transgene (T_0_) lines were generated. Among them, three were PCR-positive for the transgene.

### Semi-quantitative and real-time RT-PCR analysis

Leaves were collected, immediately frozen in liquid N_2_ and stored at −80°C. A total RNA kit (Invitrogen) was used to isolate total RNA from the stored leaves, which was then treated with RNase-free DNase (Promega) to remove genomic DNA. M-MLV reverse transcriptase (Takara) was used to synthesize first-strand cDNA. A tomato *ACTIN* fragment amplified with Actin-RT primers was used as an internal control. The primer sequences are listed in Additional file 1: Table S1.

### POD activity assays

Leaves (0.3 g), which were harvested from plants after cold treatment, were ground with 9 ml of ice-cold 20 mM KH_2_PO_4_ buffer. The homogenates were centrifuged at 4000 g at 4°C for 15 min and supernatants were used to determine enzymatic activity. POD activity was assayed by measuring the increase in absorbance at 470 nm for 3 min.The assay mixture (3 ml final volume) comprised 100 mM potassium phosphate buffer (pH 6.0), 3.7 mM H_2_O_2_, 5.0 mM guaiacol and 1 ml enzyme extract [40]. POD activity 1 U means “the change of OD_470nm_ per minute per gram fresh weight”. Each value is mean ± SE of at least ten biological replicates.

### Proline content measurement

Proline contents were determined according to [41], with some modifications. Leaves (approximately 0.3 g) were heated for 5 min in 5 mL 3% (*v*/v) aqueous acetylsalicylic acid. After cooling, the homogenate was filtered. The filtrate was mixed with glacial acetic acid, deionized water (2 ml each) and acid-ninhydrin agent (4 mL) and heated for 1 h at 100°C. The reaction mixture was extracted with 4 mL toluene. Absorbance at 520 nm was used to calculate the proline content. 1 μg/mL, 2.5 μg/mL, 5 μg/mL, 10 μg/mL, 15 μg/mL, 20 μg/mL purified proline were used as standards to produce calibration curve. Each value is mean ± SE of at least ten biological replicates.

### Enzyme assay and sugar measurement

Cell wall, vacuolar, and cytoplasmic invertase activities and sugar levels were assayed as described by Jin et al. [18]. Each value is mean ± SE of at least three biological replicates.

### ABA content measurement

An Abscisic acid immunoassay detection kit (Sigma PGR1) was used to quantitate the levels of ABA in tomato. Each value is mean ± SE of at least four biological replicates.

## Results

### Cell wall invertase activity correlates with tomato cold tolerance

**Fig. 1:**
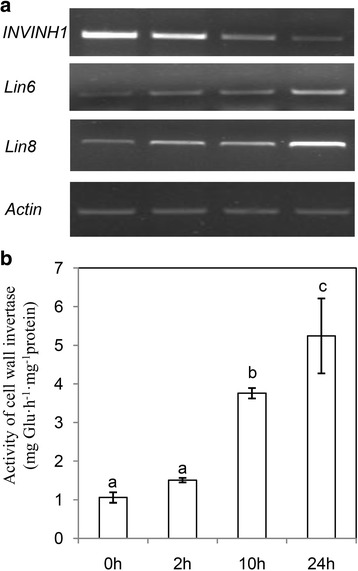
Cold suppressed *INVINH1* expression, induced cell wall invertase gene expression, meanwhile increased the activity of cell wall invertase in tomato seedlings. **a** RT-PCR analysis the expression of cell wall invertase gene (*Lin6* and *Lin8*) and cell wall invertase inhibitor gene (*INVINH1*). Expression of actin gene (*actin*) was used as an internal control. **b** Activity of cell wall invertases was progressively induced by cold treatment. Each value is mean ± SE of at least four biological replicates. Lowercase letters indicate values significantly different at *P* < 0.05

Previous research cloned the tomato cell wall invertase inhibitor (INVINH1, accession number: AJ010943) and showed that the expression of *INVINH1* was induced by ABA [18], an abiotic stress-related hormone. To determine the function of cell wall invertase and its inhibitor during tomato chilling tolerance, we compared invertase gene expression and activity of 10-d tomato seedlings before and after cold stress. The expressions of the only two known cell wall invertase genes (*Lin6* and *Lin8*) in tomato seedling were induced after treatment at 4°C for 2 h; by contrast, the expression of the cell wall invertase inhibitor gene (*INVINH1*) was depressed (Fig. 1a). Enzyme assays revealed that, compared with untreated plants, apoplastic invertase activity increased by about 150% after treatment at 4°C for 2 h and by about 520% after treatment for 24 h (Fig. 1b). These results suggest that cell wall invertase might be involved in chilling tolerance of tomato.

### Silencing *INVINH1* in tomato enhances chilling tolerance

**Fig. 2:**
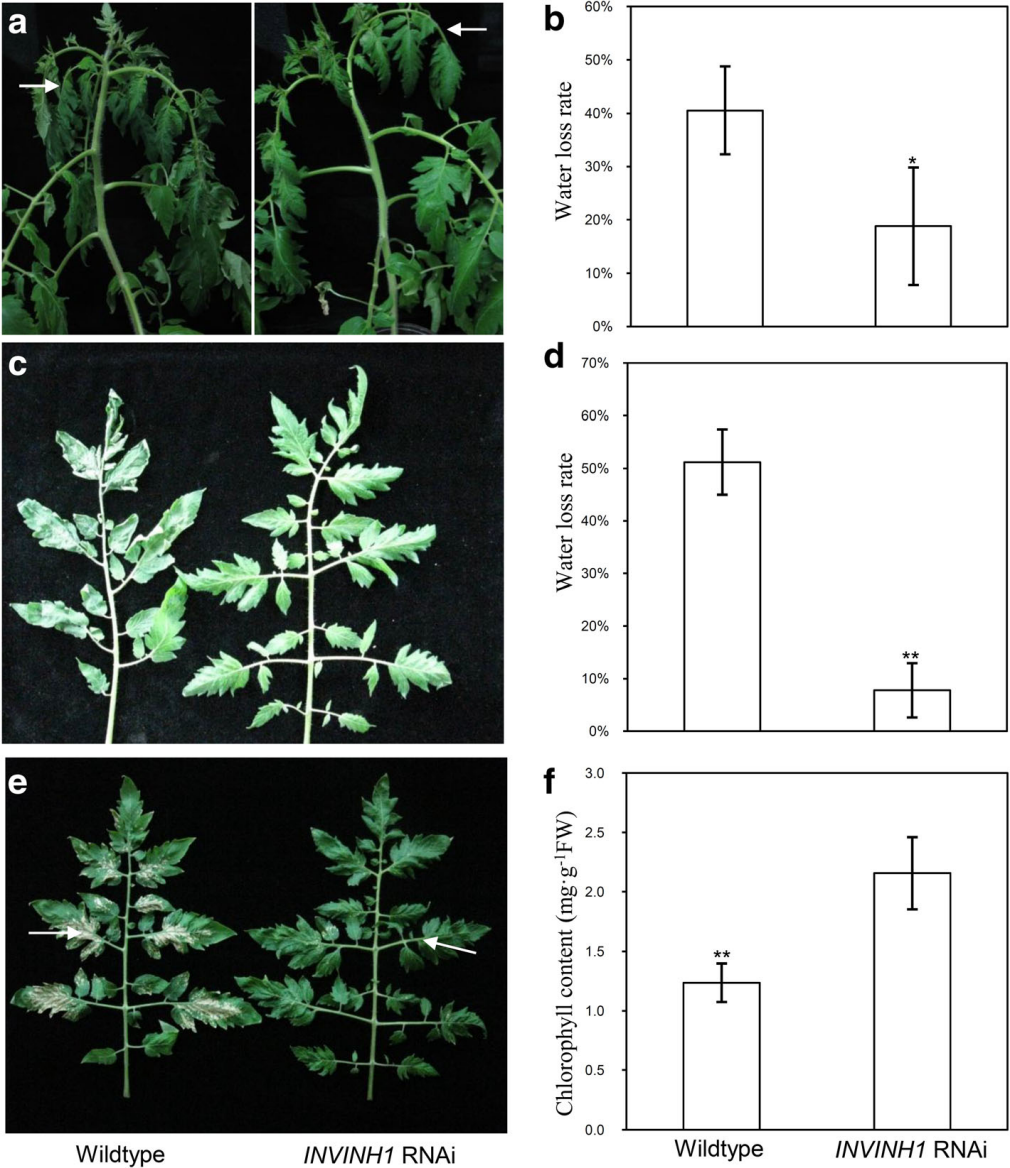
*INVINH1* silencing-expression enhanced chilling tolerance of tomato. **a** 45-d *INVINH1* RNAi and wildtype plant were treated at 4 °C for 10 h. The wildtype plant wilted, while *INVINH1* RNAi plant remained normal. **b** Water loss rate of the first and second mature leaves from (**a**). **c** The first mature leaf from 60-d *INVINH1* RNAi and wildtype plant, which were recovered at 25 °C for 2 h after treated at 4 °C for 48 h. The leaf at this position of *INVINH1* RNAi plant returned to normal at this time. **d** Water loss rate of the leaves from (**c**). **e** The third mature leaf from 60-d *INVINH1* RNAi and wildtype plant, which were recovered at 25 °C for 120 h after treated at 4 °C for 48 h. The leaf at this position of wildtype plant turned white. By contrast, the leaf at the same position of *INVINH1* RNAi remained green at this stage. **f** The chlorophyll content of the leaves from (**e**). Each value in (**b**), (**d**) and (**f**)is mean ± SE of at least ten biological replicates. An asterisk indicates a significant difference (*t* test, *P < 0.05; **P < 0.01)

In our earlier experiment, we silenced the expression of *INVINH1* in tomato by transforming an RNA interference (RNAi) construct and obtained three T_2_ homozygous transgenic lines [18]. To determine the roles of the cell wall invertase inhibitor during tomato cold tolerance, 45-day-old and 60-day-old *INVINH1* RNAi plants were treated at 4°C independently. After treated at 4°C for 10 h, the top leaves of 45-day-old wildtype plants were already wilting, but the counterpart of RNAi plants were not obviously affected (Fig. 2a & b). As for 60-day-old plants, 48 h of 4°C treatments wilted both the RNAi and wildtype plants. However the RNAi plants recovered earlier than the wildtype plants when transferred to 25°C. As shown in Fig. 2c & d, h recover at 25°C restored the first mature leaf to normal phenotype, while the leaf at the same position in the wildtype plant remained wilted. After recovered at 25°C for 5 days, both *INVINH1* RNAi and wildtype plants recovered. However, the third mature leaf from the top of 60-day-old wildtype plants was notably injured and the base of the leaf turned white, while the same position of the leaf of the *INVINH1* RNAi plant was not obviously affected at this stage (Fig. 2e & f).

**Fig. 3:**
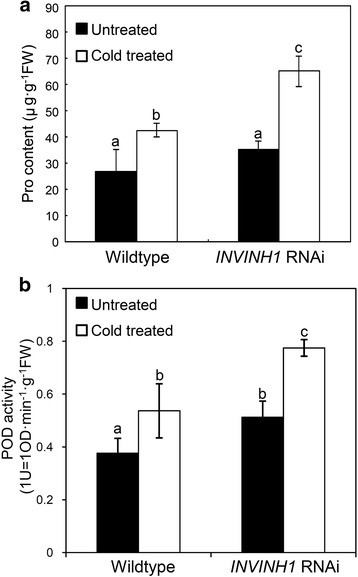
Proline content and the peroxidase (POD) activities in wildtype and *INVINH1*-scilencing plants under cold stresses. **a** The proline content of the first mature leaf from *INVINH1* RNAi and wildtype plant before and after treated at 4 °C for 24 h. **b** The POD activity of the first mature leaf from *INVINH1* RNAi and wildtype plant before and after treated at 4 °C for 24 h. (POD activity: 1 U = OD·min^−1^·g^−1^FW). Each value in (**a**) is mean ± SE of ten biological replicates. Each value in (**b**) is mean ± SE of fifteen biological replicates. Lowercase letters indicate values significantly different at P < 0.05

The accumulation of reactive oxygen species (ROS) is one of the major factors leading to chilling injury. Plant cells scavenge excess ROS using complex antioxidant system including peroxidase (POD). Proline is a known protector in abiotic stress and oxidative damage. Hsieh et al. [42] reported that the proline level is elevated in the cold insensitive transgenic tomato overexpressing *CBF1*. The activation of peroxidase (POD) and proline synthesis was used as biomarkers of chilling stress in tomato [43–45]. So we measured POD activity and proline content. 4°C treatment induced proline accumulation and peroxidase (POD) activities in both *INVINH1* RNAi and wildtype plants. After 24 h of cold treatment, the levels of proline and activities of POD were significantly lower in the wildtype than in *INVINH1* RNAi plants (Fig. 3a & b). Compared with untreated leaves, transformation with the *INVINH1* RNAi construct increased proline content by an average of 85.5% and POD activity by 51.2% after cold treatment. By contrast, wildtype plants increased their proline content by an average of 58.9% and their POD activity by 42.5% after cold treatment. These results indicate that wildtype tomato plants are more sensitive to low temperature than *INVINH1* RNAi plants. The other two *INVINH1* RNAi lines (line 2&8) showed similar results to line 1 as presented in Additional file 1: Figure S1.

### *INVINH1* overexpression tomatoes are more sensitive than wildtype plants to chilling stress

To further examine the role of *INVINH1* in tomato cold tolerance, we transformed tomato plants with an *INVINH1* overexpression construct. In our earlier experiment, we introduced a 35S:INVINH1 overexpression construct into tomato. Unfortunately, all transgenic seeds aborted and no T1 progeny were obtained [18]. To solve this problem, the Rbsc3A promoter, which does not have transcriptional activity in tomato seeds [39], was used to replace the 35S promoter in the overexpression construct. The Rbsc3A:INVINH1 overexpression construct was introduced into tomato through *Agrobacterium tumefaciens*-mediated transformation. Three primary transgenic lines (T0) were identified. The T0 plants were self-pollinated for seeds. T1 generations were analyzed to identify the presence or absence of the transgene by PCR [46]. Three T1 lines were used for detailed analysis along with their non-transgenic segregants, which were analogous to the wildtype.

**Fig. 4:**
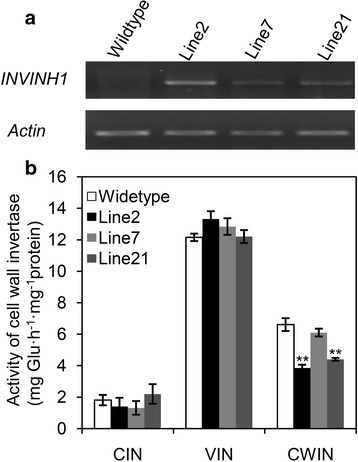
Over-expression *INVINH1* in tomato significantly depressed cell wall invertase activity. **a** RT-PCR analysis revealed *INVINH1* expression was increased in the mature leaves of *INVINH1* over-expression plants. The *actin* gene was used as an internal control. **b** Cell wall invertase (CWIN) activity of transgenic line2 & 21 was reduced significantly in the mature leaves in comparison with wildtype plant (*t* test *p* < 0.01). While the activity of cytoplasmic (CIN) and vacuolar invertases (VIN) were not affected. Each value in (**b**) is mean ± SE of at least three biological replicates. An asterisk indicates a significant difference (*t* test, **P < 0.01)

RT-PCR analyses showed that the transcription of *INVINH1* was significantly increased in the three transgenic lines, compared with the wild-type (Fig. 4a). Notably, among the three lines, line 2 showed the maximum level of *INVINH1* transcription (Fig. 4a). The overexpression of *INVINH1* led to a significant decrease in cell wall invertase activity in line 2 & 21. Especially in line 2 the transcription increase of *INVINH1* led to 42% decrease in cell wall invertase activity (Fig. 4b). Thus this was used for further analysis. No alteration of cytoplasmic or vacuolar invertases activities between transgenic and wild-type plants were detected in transgenic lines (Fig. 4b).

**Fig. 5:**
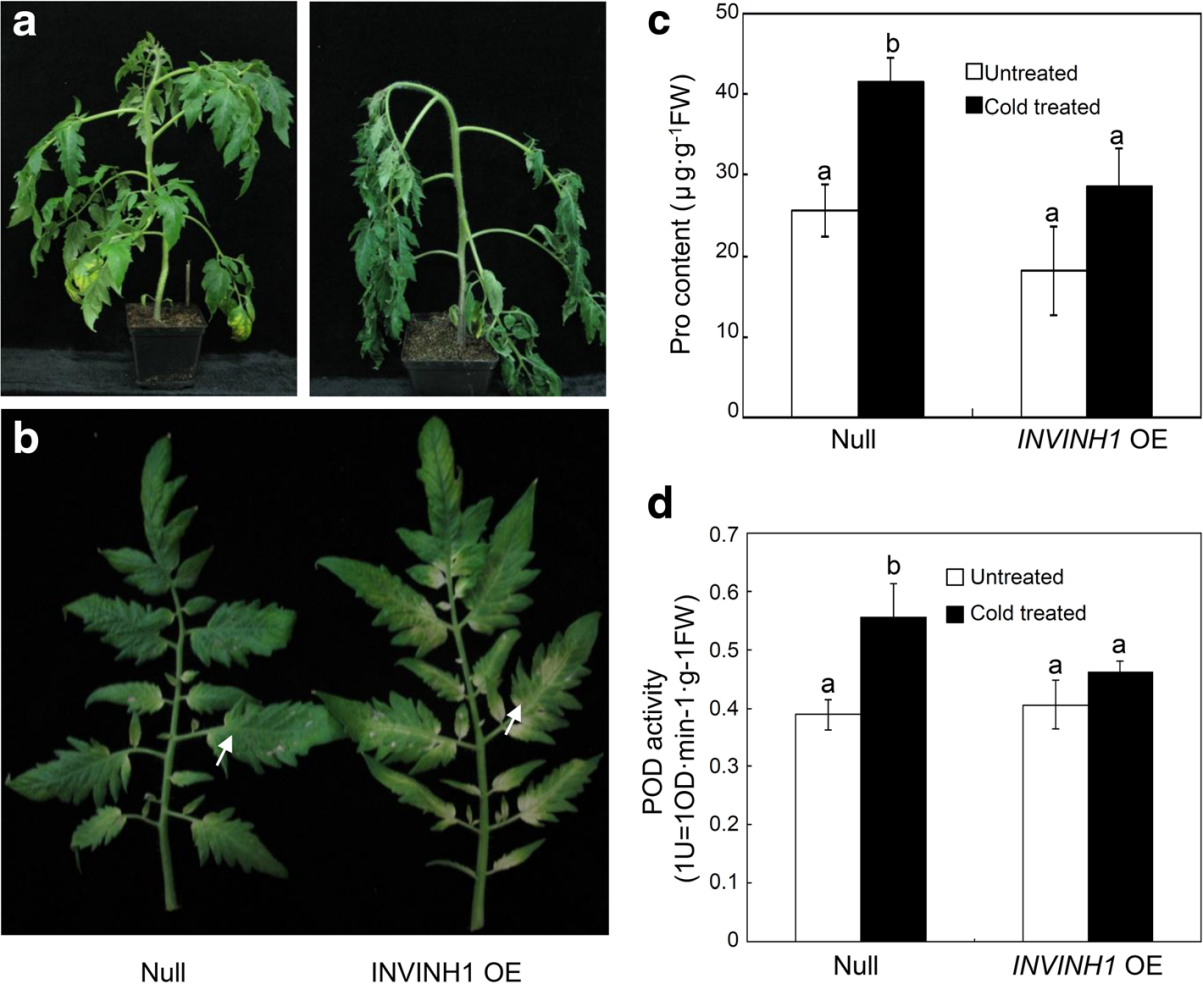
Phenotypic responses of *INVINH1* over-expression and wildtype under cold stress. **a** 45-d *INVINH1* over-expression and wildtype plant were treated at 4 °C for 6 h. The *INVINH1* over-expression plant wilted, while wildtype plant remained normal. **b** The first mature leaf from 60-d *INVINH1* RNAi and wildtype plant, which were recovered at 25 °C for 72 h after treated at 4 °C for 24 h. The leaf at this position of *INVINH1* over-expression plant turned white. By contrast, the leaf at the same position of wildtype remained green at this stage. **c** The proline content of the first mature leaf from *INVINH1* over-expression and wildtype plant before and after treated at 4 °C for 24 h. **d** The POD activity of the first mature leaf from *INVINH1* over-expression and wildtype plant before and after treated at 4 °C for 24 h. (POD activity: 1 U = OD·min^−1^·g^−1^FW). Each value in (**c**) and (**d**) is mean ± SE of at least ten biological replicates. Lowercase letters indicate values significantly different at *P* < 0.05

We exposed 45-day-old *INVINH1* overexpression and wildtype plants to 4°C. Figure 5a shows that the *INVINH1* overexpression plant was injured after exposure to 4°C for 6 h, but the wildtype was not obviously affected. Both 60-day-old *INVINH1* overexpression and wildtype plants recovered when revived at 25°C for 3 days after cold treatment. However, the first mature leaf from the top of the *INVINH1* overexpression plant was notably injured and the base of the leaf turned white, while the same position on the leaf of the wildtype plant was not obviously affected at this stage (Fig. 5b). The phenotypic responses of *INVINH1* over-expression line 21 were similar to line 2 (Additional file 1: Figure S2).

Consistent with the previous results for *INVINH1* RNAi plants, 4°C treatment induced proline accumulation and peroxidase (POD) activities in both *INVINH1* overexpression and wildtype plants. After 24 h of cold treatment, the levels of proline and activities of POD were significantly higher in wildtype compared with the *INVINH1* overexpression plants (Fig. 5c & d). In comparison with untreated leaves, transforming with *INVINH1* overexpression construct increased the proline content by an average of 57.7% and POD activity by 13.7%. In contrast, the wildtype plant increased its proline content by an average of 61.7% and its POD activity by 42.8%. These results indicated that *INVINH1* overexpression plants are more sensitive than wildtype plants to low temperature.

### Regulation of *Lin6* & *INVINH1* gene expression and sugar content in *INVINH1* overexpression and silenced plants under chilling stress

**Fig. 6:**
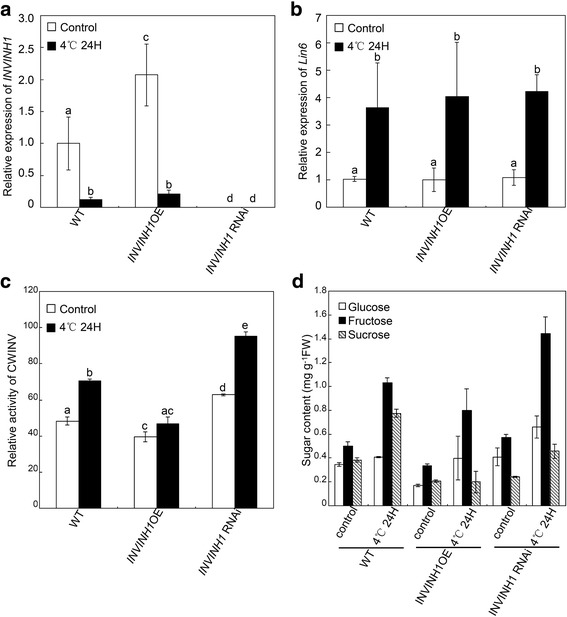
The cell wall invertase activity and hexose content were induced by chilling stress and regulated by INVINH1. **a** Expression of *INVINH1* in the first mature leaves of *INVINH1* over-expression, wildtype and RNAi plants before and after treated at 4 °C for 24 h. *Actin* was used as the loading control. **b** Expression of cell wall invertase gene *Lin6* in the first mature leaves of *INVINH1* over-expression, wildtype and RNAi plants before and after treated at 4 °C for 24 h. *Actin* was used as the loading control. **c** The activity of cell wall invertase in the first mature leaves of *INVINH1* over-expression and RNAi plants before and after treated at 4 °C for 24 h. **d** Sugar levels in the first mature leaves of *INVINH1* over-expression, wildtype and RNAi plants before and after treated at 4 °C for 24 h. Each value in (**a**), (**b**), (**c**) and (**d**) is mean ± SE of at least four biological replicates. Lowercase letters indicate values significantly different at P < 0.05

To explore the mechanism of how INVINH1 regulates cold tolerance in tomatoes, the expressions of the cell wall invertase gene *Lin6* and *INVINH1* were analyzed using real-time RT-PCR. The results revealed that the expression of *INVINH1* was depressed by cold treatment in both *INVINH1* overexpression and wildtype plants. The *INVINH1* transcript was barely detected in *INVINH1* silenced plants (Fig. 6a). The mRNA level of cell wall invertase *Lin6* was not affected by silencing or overexpression of the invertase inhibitor. Cold treatment increased the transcript levels of *Lin6* in both transgenic (*INVINH1* overexpression and RNAi) and wildtype (Fig. 6b). As a result, the apoplastic invertase activity increased after treatment at 4°C for 24 h in both transgenic (*INVINH1* overexpression and RNAi) and wildtype. Notably, although application of cold treatment increased the transcript levels of the cell wall invertase in *INVINH1* overexpression plants, their apoplastic invertase activity was still lower than those in wildtype with or without cold treatment (Fig. 6c).

Sucrose increases in wildtype and *INVINH1* RNAi under cold stress (Fig. 6d). Sugar measurement revealed increases in glucose and fructose levels after treatment at 4°C for 24 h in both transgenic (*INVINH1* overexpression and silencing) and wildtype. The hexoses content increased when the expression of *INVINH1* was silenced, before and after cold treatment. Consistently, the glucose and fructose levels decreased if the expression of *INVINH1* increased, before and after cold treatment (Fig. 6d).

### External glucose rescued the different phenotype caused by the alteration expression of *INVINH1* under chilling stress

The increase of sugar (glucose, fructose and sucrose) content by chilling stress, the increase or decrease in glucose and fructose content by silencing or overexpression of the cell wall invertase inhibitor INVINH1 and the resultant impact on cold tolerance in tomato indicated that tomato cold resistance is sensitive to sugar levels in vivo. If this is the case, exogenously applied sugars might have a positive impact on tomato cold tolerance. In consideration of glucoses not only act as sugars but also as signaling factor [47] and when external glucose supplied, the internal glucose, fructose and sucrose content accumulated in the same proportion [48], we hypothesized that exogenously applied glucose might contribute to chilling stress tolerance.

**Fig. 7:**
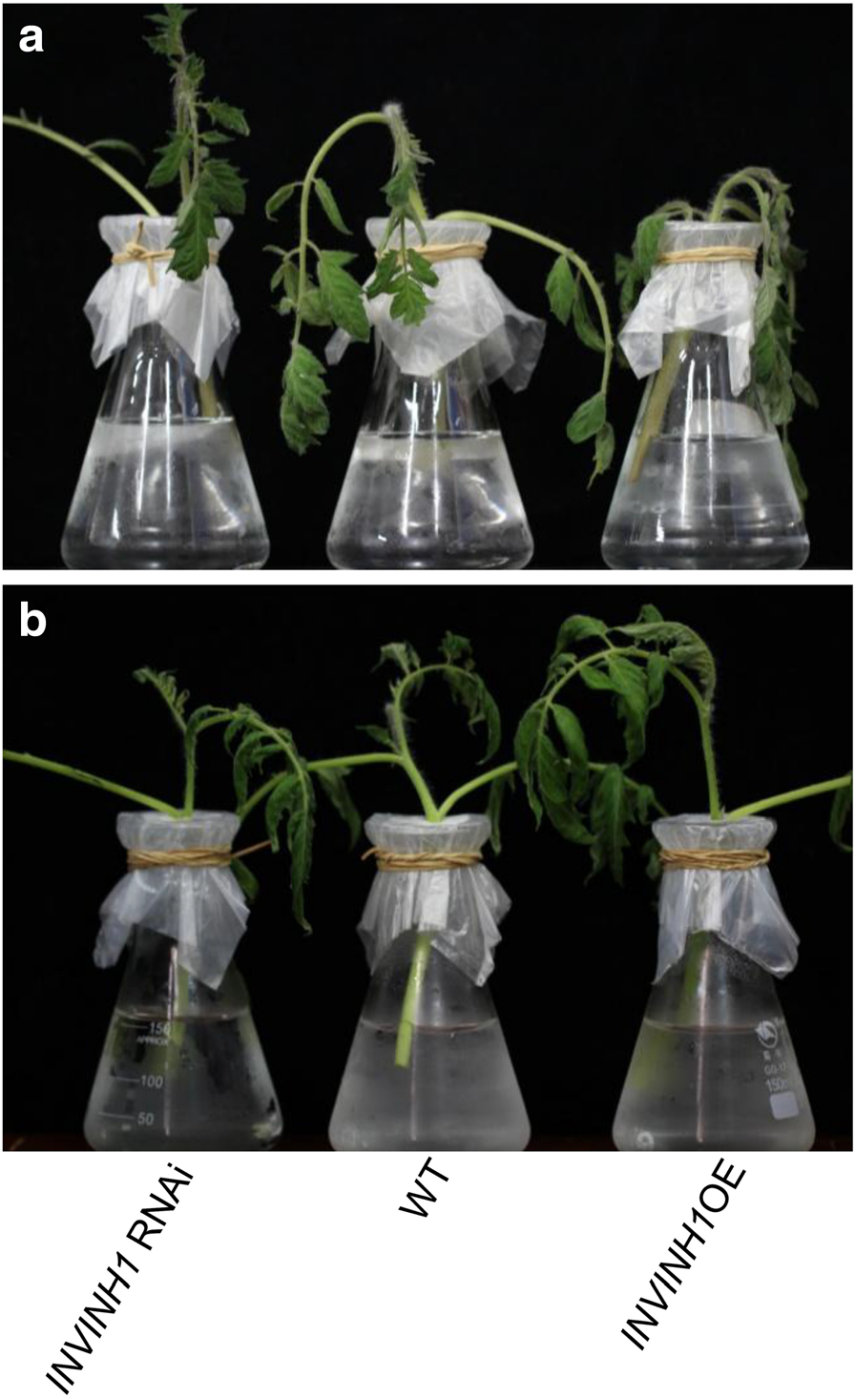
Exogenous supply of glucose applied hided the differences of chilling tolerance in tomato shoots, which were caused by the different expression of *INVINH1*. **a** The shoots from the same position of 60-d *INVINH1* RNAi, INVINH1 over expression and wildtype plant were immediately cut under water with 2% mannitol then treated at 4 °C of 24 h. **b** The shoots from the same position of 60-d *INVINH1* RNAi, INVINH1 over expression and wildtype plant were immediately cut under water with 2% glucose then treated at 4 °C of 24 h

To test this hypothesis, we chose shoots at the same stage from the same position of the transgenic and wildtype plants and treated them at 4°C for 24 h with applied 2% glucose or 2% mannitol in vitro. The results revealed that, similar to that observed above (Figs. 2 and 5), compared with the wildtype, the transgenic shoots overexpressing *INVINH1* were sensitive to cold stress, and the transgenic shoots in which the expression of *INVINH1* was silenced were relatively insensitive to chilling stress (Fig. 7a). However, when 2% glucose was added, these differences disappeared (Fig. 7b). These results suggested that the roles of INVINH1 in the cold tolerance response are overruled by high glucose level.

### Alteration of CBFs expression in *INVINH1* overexpression and silenced plants under chilling stress

**Fig. 8:**
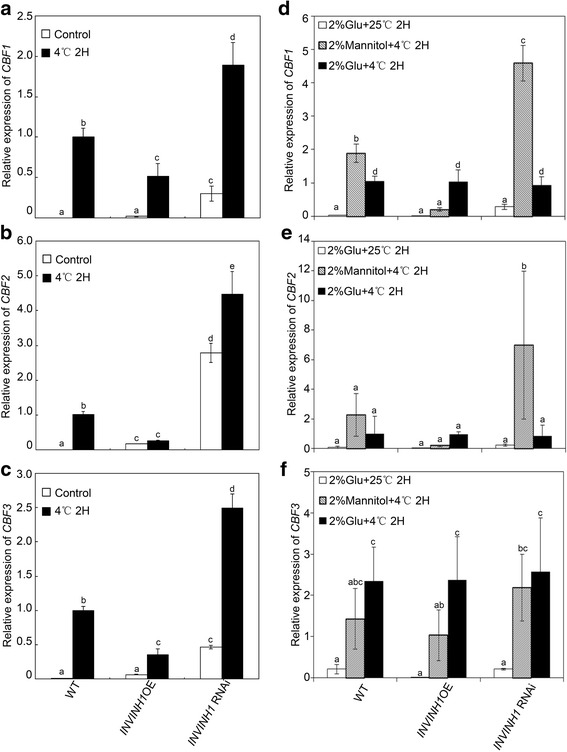
Regulation of CBFs gene expression in wildtype, INVINH1 over-expression and RNAi plants. **a** *CBF1* expression in wildtype, *INVINH1* over-expression and RNAi plants before and after cold stress. The plants were stress-treated at 4 °C for 2 h. **b** *CBF2* expression in wildtype, *INVINH1* over-expression and RNAi plants before and after cold stress. The plants were stress-treated at 4 °C for 2 h. **c** *CBF3* expression in wildtype, *INVINH1* over-expression and RNAi plants before and after cold stress. The plants were stress-treated at 4 °C for 2 h. **d** qRT-PCR analysis of the expression profiles of *CBF1* from the first mature leaf of in wildtype, *INVINH1* over-expression and RNAi plants treated by 2% glucose or 2% glucose and chilling stress or 2% mannitol and chilling stress for 2 h. **e** qRT-PCR analysis of the expression profiles of *CBF2* from the first mature leaf of in wildtype, *INVINH1* over-expression and RNAi plants treated by 2% glucose or 2% glucose and chilling stress or 2% mannitol and chilling stress for 2 h. **f** qRT-PCR analysis of the expression profiles of *CBF3* from the first mature leaf of in wildtype, *INVINH1* over-expression and RNAi plants treated by 2% glucose or 2% glucose and chilling stress or 2% mannitol and chilling stress for 2 h. Each value in (**a**), (**b**), (**c**), (**d**), (**e**) and (**f**) is mean ± SE of at least four biological replicates. *Actin* was used as the loading control. Lowercase letters indicate values significantly different at P < 0.05

To gain more insight into the role played by *INVINH1* in the cold response, we monitored the expression of cold-responsive genes by real-time PCR analysis. CBFs are known to be involved in cold tolerance [49]. Cold induced the expression of *CBFs* in wildtype (Fig. 8a–c). We examined whether the expression levels of the CBFs were altered in the *INVINH1* RNAi or overexpression plants. There was a significant reduction in transcript levels of *CBF1*, *CBF2*, and *CBF3* in the *INVINH1* overexpression plants 2 h after 4°C treatment (Fig. 8a–c). The expressions of *CBF1*, *CBF2*, and *CBF3* were consistently increased in *INVINH1* RNAi plants after treatment at 4°C for 2 h (Fig. 8a–c). This suggests that INVINH1 affects cold stress tolerance by controlling CBFs transcription.

Glucose applied in vitro masked the differences in chilling tolerance of tomato caused by the different expressions of *INVINH1* (see above) indicate that the transcription levels of *CBFs* genes may be regulated by exogenously applied hexoses. To this end, we examined expression of *CBFs* with or without 2% glucose in chilling tolerance, 2% mannitol was used as an osmotic control [50, 51]. Mannitol treatment did not affect the expression of CBFs (Fig. 8a–c and d–e). Figure 8d, e & f showed that the transcripts of these three CBFs genes were significantly up-regulated after a 2-h chilling stress with 2% glucose or mannitol. However, no difference was detected in their mRNA levels between the transgenic plants and wildtype after exposure to 4°C for 2 h with 2% glucose. These results show that INVINH1 regulated the expression of CBFs gene by adjust the contents of glucose. Interestingly compared with mannitol, glucose only induced the expression of *CBF3*. These suggest that except by adjusting the contents of glucose, INVINH1 may have another inhibitory effect on cold tolerance.

### INVINH1 suppressed endogenous ABA synthesis

ABA is an important signal in molecule plants’ response to stresses, including cold, drought and salinity [52]. The application of ABA led to a strong increase of cell wall invertase inhibitor NtCIF mRNA in tobacco [23, 53]. ABA up-regulate the expression of cell wall invertase inhibitor INVINH1 and down-regulated the expression of cell wall invertase gene lin6 in tomato [18]. Notably, exogenous glucose specifically increased the expressions of ABA synthesis and signaling genes [54]. These observations prompted us to examine whether INVINH1 affects cold stress tolerance by regulating ABA synthesis.

**Fig. 9:**
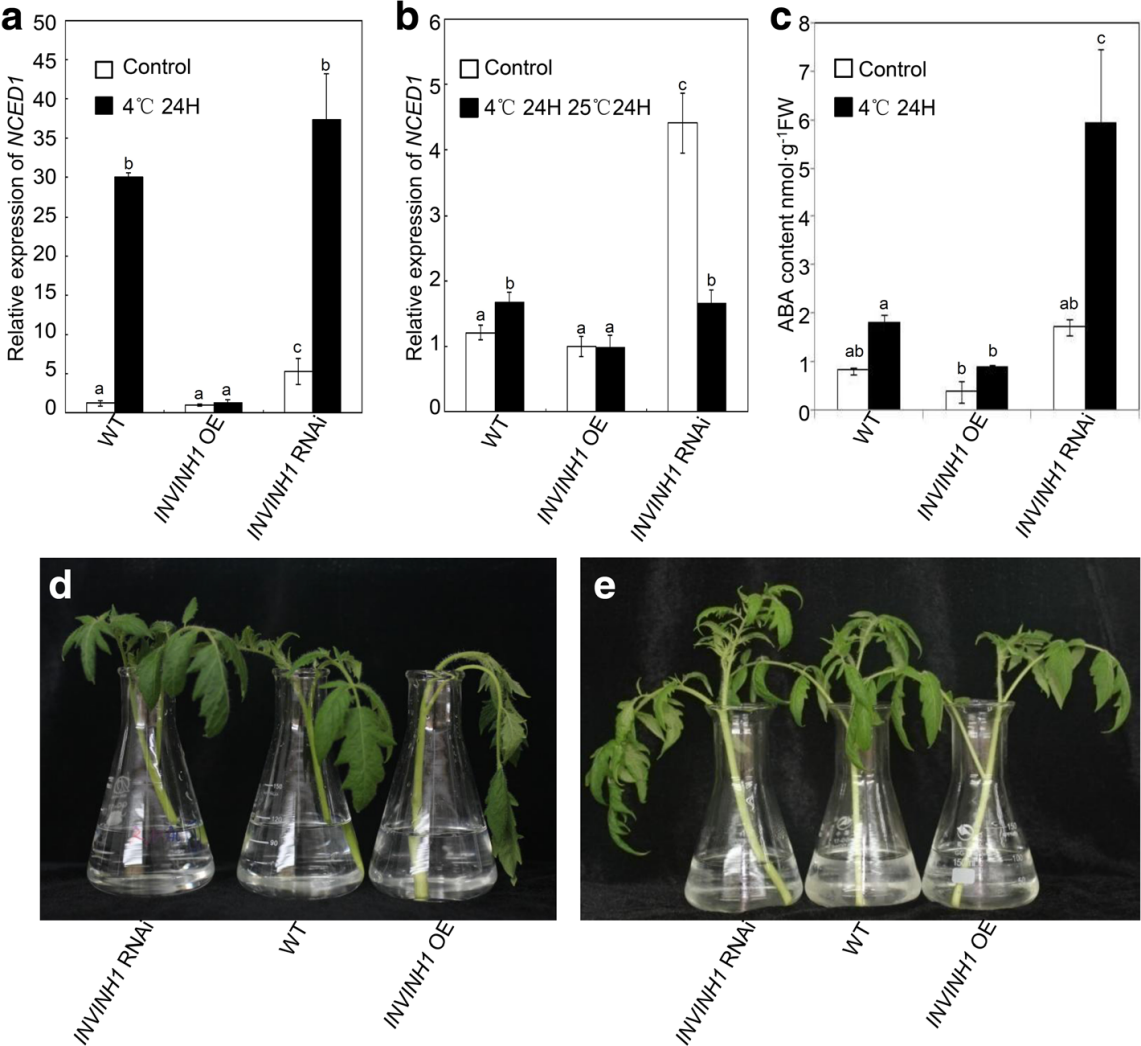
Regulation of *NCED1* gene expression in *INVINH1* over-expression and RNAi plants. **a** *NCED1* expression in *INVINH1* over-expression and RNAi plants before and after treated at 4 °C for 24 h. *Actin* was used as the loading control. **b** *NCED1* expression in *INVINH1* over-expression and RNAi plants before and after cold stress. The plants were stress-treated at 4 °C for 24 h and then recovered at 25 °C for 24H. *Actin* was used as the loading control. **c** Endogenous ABA levels in the first mature leaves of *INVINH1* over-expression, wildtype and RNAi plants before and after treated at 4 °C for 24 h. **d** The shoots from the same position of 60-d *INVINH1* RNAi, *INVINH1* over expression and wildtype plant were immediately cut under water then treated at 4 °C of 24 h. **e** The shoots from the same position of 60-d *INVINH1* RNAi, *INVINH1* over expression and wildtype plant were immediately cut under water with 10 μM ABA then treated at 4 °C of 24 h. Each value in (**a**), (**b**) and (**c**) is mean ± SE of at least four biological replicates. Lowercase letters indicate values significantly different at P < 0.05

Environmental stress regulation of ABA biosynthesis primarily occurs at the transcriptional level [10]; therefore, we examined the expression of *NCED1*, which encodes 9-cisepoxycarotenoid dioxygenase (NCED), a key enzyme in the biosynthesis of ABA [55]. Quantitative RT-PCR analysis showed that before cold treatment, there was an increase in the expression levels of *NCED1* in *INVINH1* RNAi plants compared with wildtype (Fig. 9a & b). After cold treatment, *NCED1* expression increased in both *INVINH1* RNAi and wildtype plants; however, the expression levels of *NCED1* in *INVINH1* RNAi plants were still higher compared with wildtype (Fig. 9a). No significant differences in *NCED1* expression were found between *INVINH1* RNAi and wildtype plants after recovery at 25°C for 24 h after treatment at 4°C for 24 h. Notably, in the *INVINH1* overexpression plants, the expression of *NCED1* was not obviously affected by low-temperature stress (Fig. 9a & b). After 24H of cold treatment, the levels of endogenous ABA content of the first mature leaves from wildtype were significantly higher compared with *INVINH1* overexpression plants and lower compared with *INVINH1* RNAi plants (Fig. 9c). Furthermore Fig. 9d & e showed that application of ABA protect plant from chilling damage caused by the different expression of *INVINH1*. These results suggested INVINH1 is probable involved in the regulation of endogenous ABA synthesis.

## Discussion

### Cell wall invertase inhibitor INVINH1 is involved in tomato chilling tolerance

Invertase inhibitor, which post-translationally regulates the activity of invertase, plays important roles in controlling fruit and seed development [18, 21, 28, 30, 31], leaves senescence [18], cold-induced sweetening of potato tubers [12, 29, 22, 33, 56], fruit set under heat stress [57] and the plant defense response [58]. However, to the best of our knowledge, little evidence has been presented showing the involvement of the expression of cell wall invertase inhibitor or the activity of cell wall invertase in plant chilling tolerance.

Our results demonstrate that high cell wall invertase activities, which are regulated by cell wall invertase inhibitor (INVINH1), play important roles in chilling tolerance of tomato. First, cold treatment affected the transcript level of cell wall invertase and its inhibitor, and induced the activity of cell wall invertase (Figs. 1 & 6). We hypothesized that repression of INVINH1 may be required for chilling tolerance. Second, the transformation of tomato with *INVINH1* RNAi constructs led to enhanced chilling tolerance of the transgenic tomatoes (Fig. 2). These data demonstrated that the presence of the *INVINH1* RNAi transgene is the causal basis of the observed enhanced chilling tolerance phenotype. By contrast, overexpression of *INVINH1* in tomatoes made them more sensitive to cold stress compared with the wildtype plants (Fig. 5).These data confirmed the role of *INVINH1* in tomato cold tolerance. Finally and importantly, the degree of *INVINH1* expression correlated well with the level of plant chilling tolerance (Figs. 6 & 7a). These results demonstrate that the tomato chilling tolerance is highly sensitive to changes in *INVINH1* expression.

### INVINH1 regulated chilling tolerance by adjusting the expression of *CBFs* genes and ABA synthesis

During cold tolerance, plants reprogram their gene expression through transcriptional, posttranscriptional and posttranslational mechanisms [59]. Among various factors, the CBF-dependent transcriptional pathway induces or is involved in cold tolerance in plants such as Arabidopsis [60], rice [61], wheat [62] and tomato [42]. In tomatoes, overexpression of Arabidopsis CBF1 gene increased chilling tolerance [42]. The underlying molecular mechanism, however, remains unclear. The expressions of CBFs were suppressed by INVINH1 after 4°C treatments (Fig. 8); therefore, we hypothesized that the repression of INVINH1 may be required for the CBF-dependent cold tolerance pathway. Silencing the expression of INVINH1 induced the expression of CBF genes, even before cold treatment (Fig. 8), indicating that the expression of INVINH1 is a prerequisite for CBF-dependent cold tolerance.

ABA accumulates during cold stress and is involved in chilling tolerance [52]. Our analyses revealed that the expression of *INVINH1* affected the transcript level of the ABA synthesis gene *NCED1* (Fig. 9). The above analyses concur with previous findings [10] that environmental stress regulation of ABA biosynthesis primarily occurs at the level of transcription; however, the expression of *INVINH1* suppressed the expression of endogenous ABA synthesis (Fig. 9).

### INVINH1 controls cell wall invertase activity, which contributes to chilling tolerance by regulating the hexose content

The glucose and fructose levels were induced after cold treatment in both wildtype and transgenic plants (Fig. 6d). These data suggested that the glucose and fructose contents need to reach optimum levels to protect the plant from the cold stress. This may be achieved by adjusting the activity of cell wall invertase, because the expression of a cell wall invertase gene (*Lin6*) was induced (Fig. 6b) and the cell wall inhibitor gene was depressed by cold treatment (Fig. 6a).

Cell wall invertase, whose activity is tightly regulated by its inhibitor *in planta* at the protein level, maintains the apoplastic glucose and fructose content at an optimum level [6]. Apoplastic glucose and fructose not only provide carbon nutrients, but also play major roles in sugar signaling. For example, in maize, mutation of a cell wall invertase (*INCW2*) resulted in a miniature seed phenotype [20, 63].In tobacco, silencing the expression of cell wall invertase led to pollen abortion [64]. Interestingly, the phenotypes above, which were caused by cell wall invertase deficiency, could be partially, but not completely, recovered by exogenous supply of hexoses [23, 65]. However, unlike the phenotype caused by cell wall invertase deficiency in reproductive tissue with rapid mitosis, in our study, the differences in chilling tolerance of tomatoes that were caused by different expressions of *INVINH1* were totally blocked by the application of 2% glucose in vitro (Fig. 7).

## Conclusions

Cell wall invertase inhibitor INVINH1 plays an important role in regulation of chilling tolerance in tomato by adjusting the sugar content, the expression of CBFs genes.

## Additional files

Additional file 1: Table S1. Primer sequences used for reverse transcriptase (RT)-PCR analysis of C-repeat binding factors (CBFs), ABA biosynthesis and signaling, and certain tomato invertase and invertase inhibitor genes. **Figure S1.** Phenotypic responses of *INVINH1* RNAi line 2&8 and wildtype plant under cold stress. (A) Water loss rate of the leaves from The first mature leaf from 60-d *INVINH1* RNAi and wildtype plant, which were recovered at 25 °C for 2 h after treated at 4 °C for 48 h. (B) The proline content of the first mature leaf from 60-d *INVINH1* RNAi and wildtype plant before and after treated at 4 °C for 24 h. (C) The POD activity of the first mature leaf from 60-d *INVINH1* RNAi and wildtype plant before and after treated at 4 °C for 24 h. (POD activity: 1 U = OD•min^−1^•g^−1^FW). Each value is mean ± SE of at least ten biological replicates. Lowercase letters indicate values significantly different at *P* < 0.05. **Figure S2.** Phenotypic responses of *INVINH1* over-expression line 21 and wildtype plant under cold stress. (A) 65-d *INVINH1* over-expression and wildtype plant were treated at 4 °C for 24 h. The *INVINH1* over-expression plant wilted, while wildtype plant remained normal. (B) The first mature leaf from 65-d *INVINH1* RNAi and wildtype plant, which were recovered at 25 °C for 72 h after treated at 4 °C for 24 h. The leaf at this position of *INVINH1* over-expression plant turned white. By contrast, the leaf at the same position of wildtype plant remained green at this stage. (DOCX 731 kb)

## Abbreviations

ABA: Abscisic acid
CBF: C-repeat binding factors
NCED1: 9-cisepoxycarotenoid dioxygenase1
OE: Overexpression
pI: Isoelectric point
POD: Peroxidase
RNAi: RNA interference
WT: Wildtype
