# Supplementary material for: The roles of call wall invertase inhibitor in regulating chilling tolerance in tomato
Source: BMC Plant Biol. 2017 Nov 9;17:195. doi: 10.1186/s12870-017-1145-9 (PMC5679139; doi:10.1186/s12870-017-1145-9)
Supplement: Supplementary file 1 — Primer sequences used for reverse transcriptase (RT)-PCR analysis of C-repeat binding factors (CBFs), ABA biosynthesis and signaling, and certain tomato invertase and invertase inhibitor genes. Figure S1. Phenotypic responses of INVINH1 RNAi line 2&8 and wildtype plant under cold stress. (A) Water loss rate of the leaves from The first mature leaf from 60-d INVINH1 RNAi and wildtype plant, which were recovered at 25 °C for 2 h after treated at 4 °C for 48 h. (B) The proline content of the first mature leaf from 60-d INVINH1 RNAi and wildtype plant before and after treated at 4 °C for 24 h. (C) The POD activity of the first mature leaf from 60-d INVINH1 RNAi and wildtype plant before and after treated at 4 °C for 24 h. (POD activity: 1 U = OD•min−1•g−1FW). Each value is mean ± SE of at least ten biological replicates. Lowercase letters indicate values significantly different at P < 0.05. Figure S2. Phenotypic responses of INVINH1 over-expression line 21 and wildtype plant under cold stress. (A) 65-d INVINH1 over-expression and wildtype plant were treated at 4 °C for 24 h. The INVINH1 over-expression plant wilted, while wildtype plant remained normal. (B) The first mature leaf from 65-d INVINH1 RNAi and wildtype plant, which were recovered at 25 °C for 72 h after treated at 4 °C for 24 h. The leaf at this position of INVINH1 over-expression plant turned white. By contrast, the leaf at the same position of wildtype plant remained green at this stage. (DOCX 731 kb) [file 12870_2017_1145_MOESM1_ESM.docx]

**Table S1**. Primer sequences used for reverse transcriptase (RT)-PCR analysis of C-repeat binding factors (CBFs), ABA biosynthesis, and certain tomato invertase and invertase inhibitor genes.

| Name | Sequence^a^ |
| --- | --- |
| CBF1 | F: 5'-TCTTCTTCATCGTCATCGTCGT-3' |
|  | R: 5'-GTAGGCATCAGTTTCCACACAAT-3' |
| CBF2 | F: 5'-ACTAATAATCTCAATCACTACTCCCC-3' |
|  | R: 5'-GCGTGCACATCAGCTAATTCA-3' |
| CBF3 | F: 5'-GGACCCACGTATAGAATCTTGTT-3' |
|  | R: 5'-ACCACTGATTCTTCTTCCTCCG-3' |
| NCED1 | F: 5'-GTATGACACCACCAGACTCCA-3' |
|  | R: 5'-AAGCTTCACAGTTGCCTCCA-3' |
| LIN6 | F: 5'-GTAGATGTAGATTTAGCAGA-3' |
|  | R: 5'-CTATGGTTTCTTTGTGACGT-3' |
| LIN8 | F: 5'-CTGGATATGTAGATGTAGAT-3' |
|  | R: 5'-ACGTCTGCTCAAATATCATG-3' |

^a^:F: forward primer; R: reverse primer.


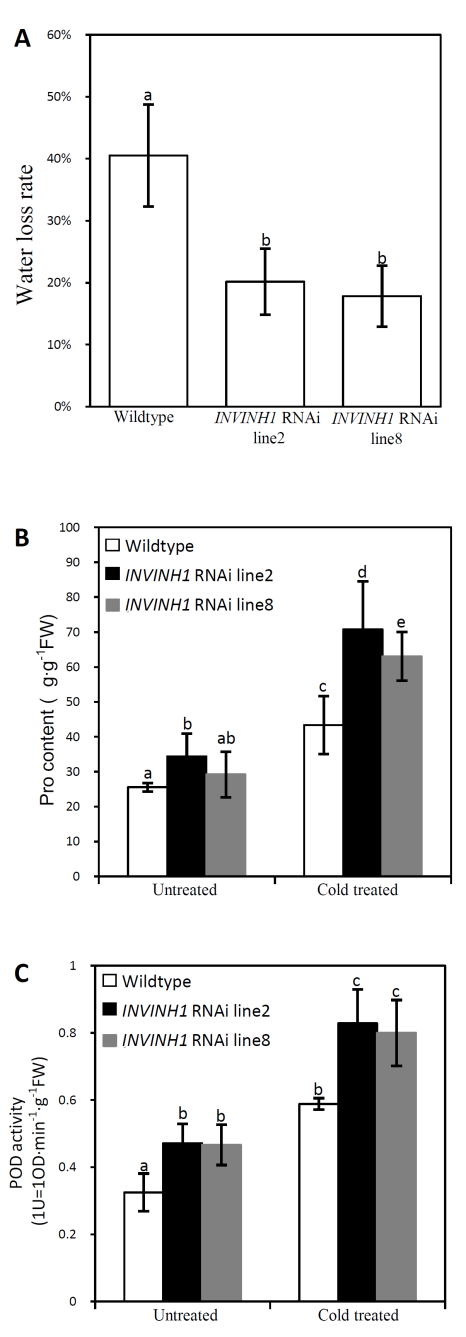


**Figure S1** Phenotypic responses of *INVINH1* RNAi line 2&8 and wildtype plant under cold stress.

(A) Water loss rate of the leaves from The first mature leaf from 60-d INVINH1 RNAi and wildtype plant, which were recovered at 25℃ for 2 hours after treated at 4℃ for 48 hours.

(B) The proline content of the first mature leaf from 60-d *INVINH1* RNAi and wildtype plant before and after treated at 4℃ for 24 hours.

(C) The POD activity of the first mature leaf from 60-d *INVINH1* RNAi and wildtype plant before and after treated at 4℃ for 24 hours. (POD activity: 1U=OD·min^-1^·g^-1^FW)

Each value is mean±SE of at least ten biological replicates. Lowercase letters indicate values significantly different at P <0.05.


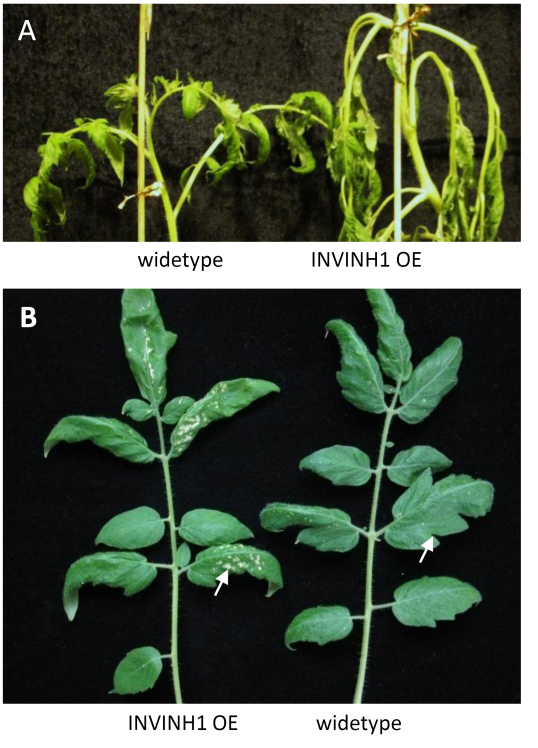


**Figure S2** Phenotypic responses of *INVINH1* over-expression line 21 and wildtype plant under cold stress.

(A) 65-d *INVINH1* over-expression and wildtype plant were treated at 4℃ for 24 hours.

(B) The first mature leaf from 65-d *INVINH1* RNAi and wildtype plant, which were recovered at 25℃ for 72 hours after treated at 4℃ for 24 hours.
